# Supplementary material for: Strain-resolved analysis of hospital rooms and infants reveals overlap between the human and room microbiome
Source: Nat Commun. 2017 Nov 27;8:1814. doi: 10.1038/s41467-017-02018-w (PMC5703836; doi:10.1038/s41467-017-02018-w)
Supplement: Supplementary file 1 — Supplementary Information [file 41467_2017_2018_MOESM1_ESM.pdf]

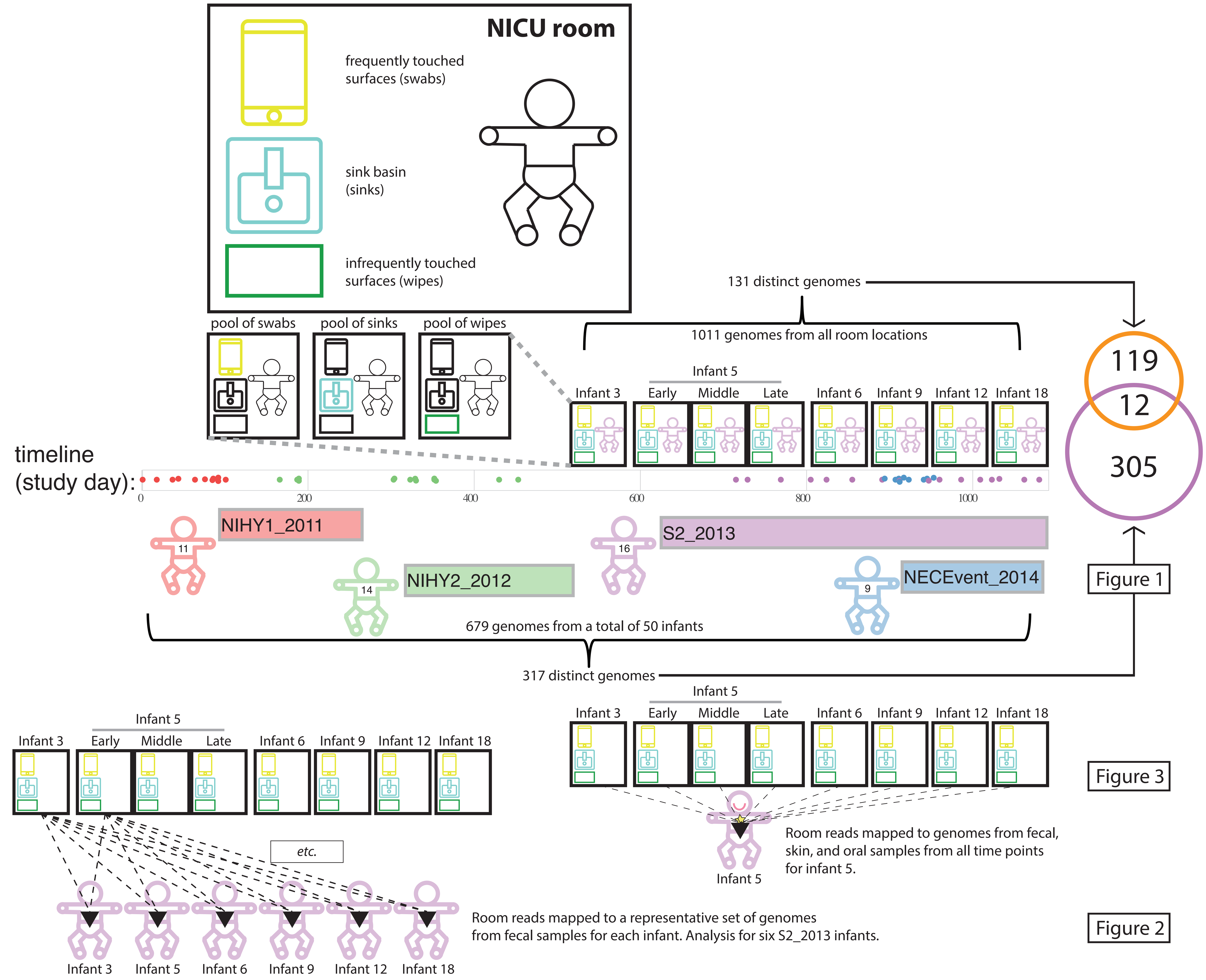

Supplementary Figure 1: Overview of our experimental approach, explaining the analyses that were used to generate Figures 1, 2 and 3. Dots in the centrally placed timeline indicate birth dates of the 50 infants involved in this study. Information above the timeline illustrates the room sampling approach and below the timeline details the infant cohort from which fecal samples were collected for genome-resolved metagenomic analysis. Analyses for Fig. 1 involved genome comparisons, whereas those for Fig. 2 and 3 involved mapping of reads to genomes reconstructed de novo from fecal samples from Infant 5 (Fig. 3) and all infants whose rooms were sampled (Fig. 2).

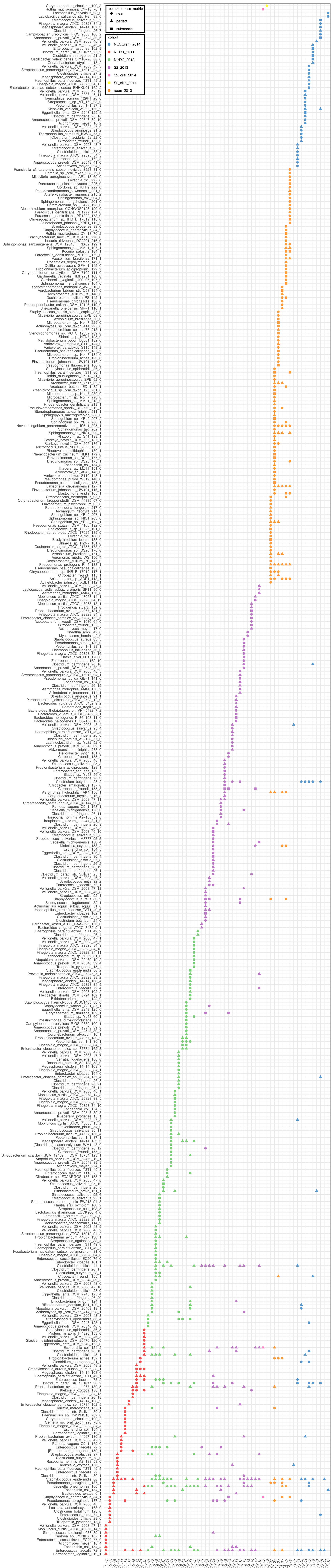

Supplementary Figure 2: Strains found in the infant gut across several cohorts and years are also present in rooms. Fecal and room samples from defined sampling time periods (the 2011, 2012, 2013 and 2014 cohorts) are arrayed on the x-axis. This plot is an expansion of Fig. 1 in that it reveals information about each infant within a cohort along the x-axis and all genomes, including genomes only found in one infant, on the y-axis. The y-axis labels indicate a representative genotype from clusters of genotypes that shared > 99% gANI. The first digit in the name indicates the species and the second indicates the specific strain. See Supplementary Data 3-11 for further genome clustering results. Shapes indicate how complete a genome is using CheckM's completion metric.

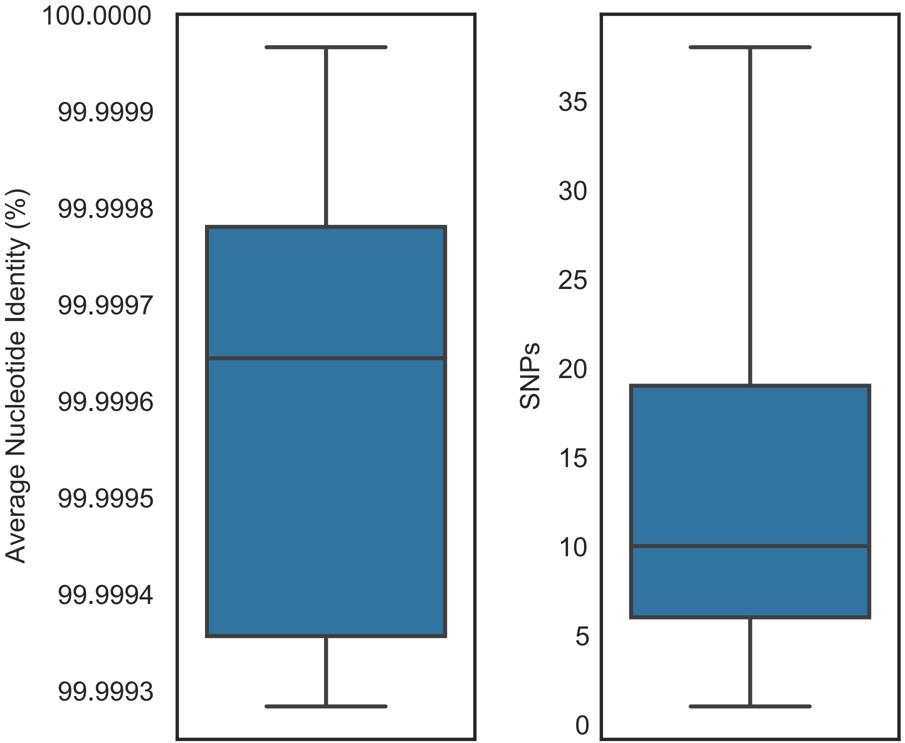

Supplementary Figure 3: Establishment of an average nucleotide identity (ANI) threshold. For infants S2_2013_005, S2_2013_006, S2_2013_009, and S2_2013_018, reads from all fecal samples were merged and mapped to the infants' dereplicated genome set. The distribution of ANI values and total SNP counts for all genomes tested is shown (outliers excluded). SNPs may result from assembly errors or non-specific mapping, and represent the limit of detection of this method.

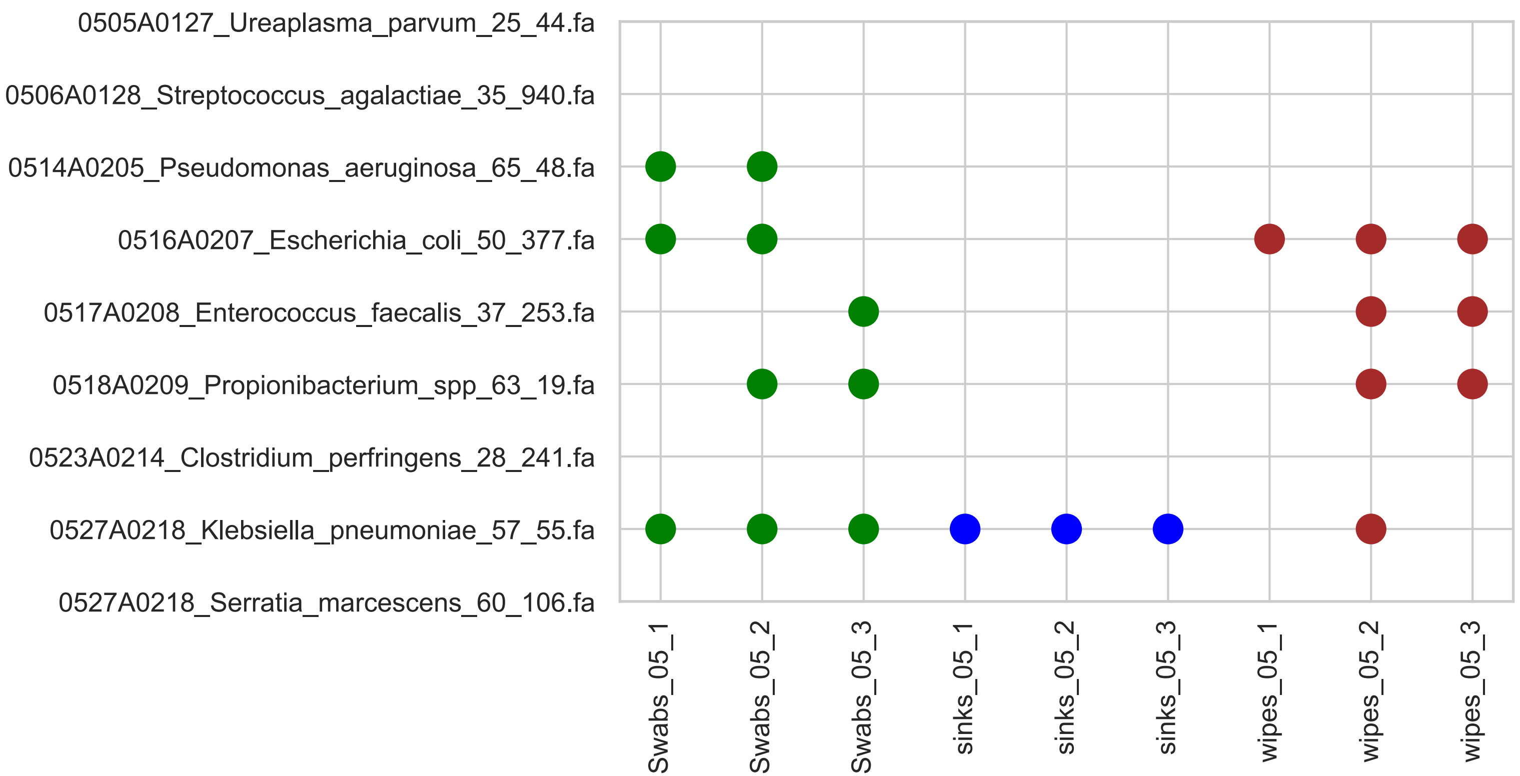

Supplementary Figure 4: Strains shared in Infant 5's room and gut time series. Reads from all room metagenomes were mapped to Infant 5's dereplicated genome set. Symbols indicate fecal genomes preset in the room with at least 99.999% ANI. Almost all strains assembled from the infant are also found in the room environment.

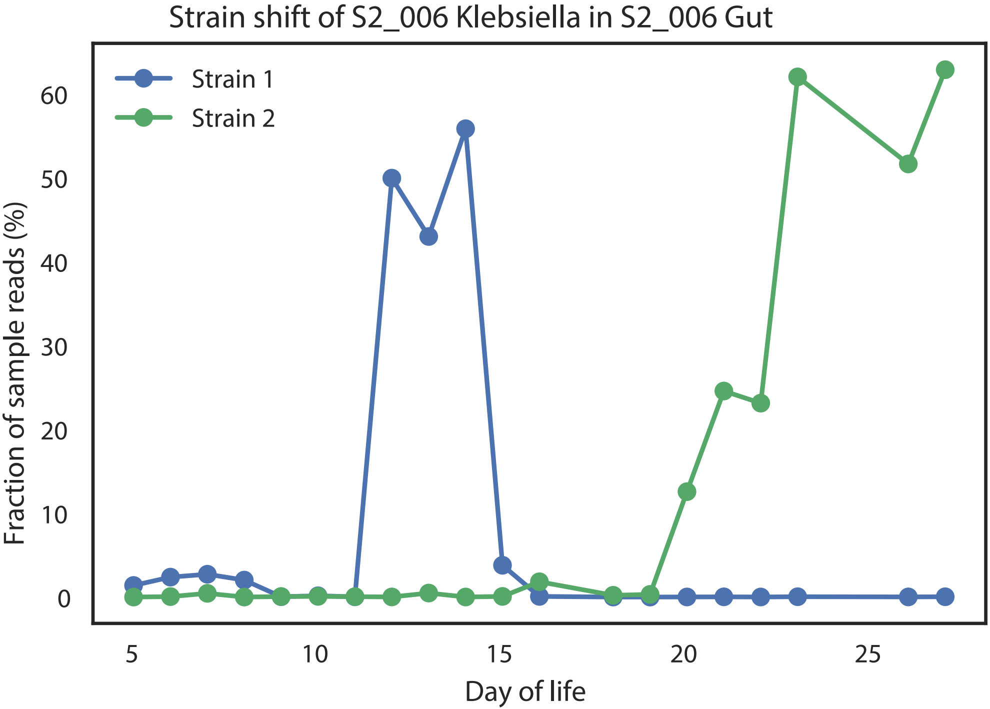

Supplementary Figure 5: A *K. pneumoniae* strain shift occurs Infant 6's fecal samples. The relative abundance of strain 1 (identified in the room environment) and strain 2 (not identified in the room environment) in infant S2_2013_006's fecal samples. Stain 2 overtakes strain 1 completely by day of life 20.
