# Supplementary material for: Strain-resolved analysis of hospital rooms and infants reveals overlap between the human and room microbiome
Source: Nat Commun. 2017 Nov 27;8:1814. doi: 10.1038/s41467-017-02018-w (PMC5703836; doi:10.1038/s41467-017-02018-w)
Supplement: Supplementary file 3 — Description of Additional Supplementary Files [file 41467_2017_2018_MOESM3_ESM.pdf]

Description of Additional Supplementary Files

File Name: Supplementary Data 1
Description: Room sample pooling.

File Name: Supplementary Data 2
Description: Infant metadata.

File Name: Supplementary Data 3
Description: Genome information from the genome de-replication workflow. This table details genome input locations, filenames, and lengths.

File Name: Supplementary Data 4
Description: Genome clustering information from the genome de-replication workflow. This table details primary cluster, secondary cluster, and information on clustering method for each genome.

File Name: Supplementary Data 5
Description: CheckM information from the genome de-replication workflow. This table details CheckM results for all genomes.

File Name: Supplementary Data 6
Description: Mash comparisons from the genome de-replication workflow. This table details pair-wise Mash comparison results.

File Name: Supplementary Data 7
Description: Average nucleotide identity results from the genome de-replication workflow. This table details secondary comparison results.

File Name: Supplementary Data 8
Description: dRep genome scores from the genome de-replication workflow. This table details the score of each genome.

File Name: Supplementary Data 9
Description: Genome taxonomy information from the genome de-replication workflow. This table provides Centrifuge derived taxonomic information for each genome.

File Name: Supplementary Data 10
Description: dRep cluster scores from the genome de-replication workflow. This table details the cluster and score of each de-replicated genome.

File Name: Supplementary Data 11
Description: Genome statistics from the genome de-replication workflow. This table details useful checkM information on de-replicated genomes.

File Name: Supplementary Data 12
Description: Room reads mapped to infant genomes. This table details all room samples mapped against all dereplicated infant genomes and run through PileupProfile (supports Fig. 2 and Supplementary Fig. 4).

File Name: Supplementary Data 13
Description: Room reads mapped to time series fecal genomes. Output from the workflow used to track presence or absence of infant gut colonizing organisms from NICU rooms via read mapping (supports Fig. 3).
